# Assessment of Anti-SARS-CoV-2 antibody levels among university students vaccinated with different COVID-19 primary and booster doses — fall 2021, Wisconsin

**DOI:** 10.1186/s12879-023-08332-7

**Published:** 2023-06-05

**Authors:** Peter M. DeJonge, Anastasia S. Lambrou, Hannah E. Segaloff, Allen Bateman, Alana Sterkel, Carol Griggs, Jake Baggott, Patrick Kelly, Natalie Thornburg, Monica Epperson, Rodel Desamu-Thorpe, Glen Abedi, Christopher H. Hsu, Jasmine Y. Nakayama, Jasmine Ruffin, Darlene Turner-Harper, Almea Matanock, Olivia Almendares, Melissa Whaley, Ayan Chakrabarti, Kyle DeGruy, Michele Daly, Ryan Westergaard, Jacqueline E. Tate, Hannah L. Kirking

**Affiliations:** 1 Epidemic Intelligence Service, CDC, Atlanta, Georgia 30329 USA; 2 Wisconsin Department of Health Services, Division of Public Health, Madison, Wisconsin 53703 USA; 3 Wisconsin State Laboratory of Hygiene, Madison, Wisconsin 53703 USA; 4 University Health Services, University of Wisconsin – Madison, Madison, Wisconsin 53703 USA; 5 CDC COVID-19 Response Team, Atlanta, Georgia 30329 USA

**Keywords:** COVID-19 vaccines, Heterologous boosters, SARS-CoV-2 serology, Meso scale discovery

## Abstract

**Background:**

University students commonly received COVID-19 vaccinations before returning to U.S. campuses in the Fall of 2021. Given likely immunologic variation among students based on differences in type of primary series and/or booster dose vaccine received, we conducted serologic investigations in September and December 2021 on a large university campus in Wisconsin to assess anti-SARS-CoV-2 antibody levels.

**Methods:**

We collected blood samples, demographic information, and COVID-19 illness and vaccination history from a convenience sample of students. Sera were analyzed for both anti-spike (anti-S) and anti-nucleocapsid (anti-N) antibody levels using World Health Organization standardized binding antibody units per milliliter (BAU/mL). Levels were compared across categorical primary COVID-19 vaccine series received and binary COVID-19 mRNA booster status. The association between anti-S levels and time since most recent vaccination dose was estimated by mixed-effects linear regression.

**Results:**

In total, 356 students participated, of whom 219 (61.5%) had received a primary vaccine series of Pfizer-BioNTech or Moderna mRNA vaccines and 85 (23.9%) had received vaccines from Sinovac or Sinopharm. Median anti-S levels were significantly higher for mRNA primary vaccine series recipients (2.90 and 2.86 log [BAU/mL], respectively), compared with those who received Sinopharm or Sinovac vaccines (1.63 and 1.95 log [BAU/mL], respectively). Sinopharm and Sinovac vaccine recipients were associated with a significantly faster anti-S decline over time, compared with mRNA vaccine recipients (*P* <.001). By December, 48/172 (27.9%) participants reported receiving an mRNA COVID-19 vaccine booster, which reduced the anti-S antibody discrepancies between primary series vaccine types.

**Conclusions:**

Our work supports the benefit of heterologous boosting against COVID-19. COVID-19 mRNA vaccine booster doses were associated with increases in anti-SARS-CoV-2 antibody levels; following an mRNA booster dose, students with both mRNA and non-mRNA primary series receipt were associated with comparable levels of anti-S IgG.

## Background

The widespread availability of coronavirus disease (COVID-19) vaccines in the United States helped enable U.S. universities to offer a return to in-person learning during the 2021–2022 academic year. Vaccination was an important component of COVID-19 mitigation policies on university campuses because of the higher transmission risk associated with congregate housing (e.g., dormitories), large group learning environments, and activity in social settings (e.g., parties, athletic events, or bars) [1–5]. During fall 2021, multiple vaccines for SARS-CoV-2, the causative virus of the COVID-19 pandemic, were available. Three vaccines had been approved or authorized for emergency use by the Food and Drug Administration, including Pfizer-BioNTech and Moderna mRNA platform vaccines, and Johnson & Johnson’s Janssen (J&J/Janssen), a vector-based platform vaccine [6]. Other vaccines were available internationally under approval by the World Health Organization (WHO), including vaccines from Sinopharm (BIBP) and Sinovac (CoronaVac), both inactivated whole virus (IWV) platform vaccines [7].

The University of Wisconsin (UW) in Madison, Wisconsin, is a large, public university in an urban setting with >45,000 students on campus every academic year. Receipt of COVID-19 vaccination was not required for students returning to campus in September 2021; however, UW reported that 88% of the student body had offered proof of completion of the full COVID-19 primary series vaccination by the first week of classes [8]. Notably, UW reported 6,480 international students enrolled during fall 2021, which suggested a student body with a mix of varying COVID-19 vaccine platforms received [9]. This might have had implications for COVID-19 mitigation on campus given the variable effectiveness against COVID-19 outcomes and different immune responses associated with various types of COVID-19 vaccines [10–16].

During the fall semester of the 2021–2022 academic year at UW, we conducted a serosurvey to measure anti-SARS-CoV-2 antibodies within this highly and heterogeneously vaccinated population. Our first investigation objective was to quantify levels of SARS-CoV-2 antibodies associated with different types of COVID-19 vaccine platforms (i.e., mRNA, vector-based, and IWV). During our investigation, the U.S. Centers for Disease Control and Prevention (CDC) recommended a booster dose of an mRNA COVID-19 vaccine for all persons aged ≥16 years. This led us to our second objective, which was to quantify effects of booster doses on antibody levels among students who had received different primary vaccine types.

## Methods

We visited the UW campus at the semester start (September 7–11, 2021) and semester end (December 7–12, 2021) during the fall semester of the 2021–22 academic year. Before both the September and December project iteration, we sent out a recruitment email to students living in UW-affiliated housing (e.g., dormitories). This email informed students about the serology project and contained a link to a questionnaire that requested self-reported demographic and COVID-19 vaccination information. If students wanted to participate in the serology project, they were required to complete the questionnaire.

Students were allowed to participate in both September and December. Demographic information collected in September included age, sex, race and ethnicity, class year, and immunocompromising health conditions (based on self-reported AIDS/HIV status, recent organ transplant, or others). For the December demographics questionnaire, we added a question about country of residence because we noted a substantial proportion of international students had participated in September.

Table 1 summarizes the dose schedule and type of COVID-19 vaccines assessed by our project. Based on UW policy, to opt out of weekly COVID-19 viral testing, students were required to report COVID-19 vaccination data to UW. UW vaccination data were routinely updated throughout the fall 2021 semester. UW provided us with these data, which included vaccination dates, dose number, and type. UW-collected student vaccination data were used to validate the self-reported vaccination information from the online questionnaire. If disagreements occurred between self-reported and UW-collected vaccination information (related to vaccine types, dose dates, or overall vaccine status) we deferred to UW-collected data. Completion of any primary series was considered fully vaccinated by UW.

**Table 1:** Summary of COVID-19 vaccine types, platforms, and dosing schedules for nonimmunocompromised domestic and international students attending the University of Wisconsin - Madison, fall 2021

**COVID-19 vaccine manufacturer**	**Vaccine Platform**	**Primary Series Dosing**	**Booster Dose Guidance,** **in United States, at time of investigation**^a^	**Alternative vaccine names**	**Anti-spike** **antibody elicited**	**Anti-nucleocapsid antibody elicited**
Covaxin	Inactivated, whole virus	2 doses, 28 days apart	n/a	BBV152	✓	✓
Covishield (AstraZeneca)	Chimp Adenovirus Vector	2 doses, 28-84 days apart	n/a	ChAdOx1-S [recombinant]	✓	
Johnson & Johnson’s Janssen (J&J/Janssen)	Human Adenovirus Vector	1 dose	*In most situations, Pfizer-BioNTech or Moderna booster are recommended over a J&J /Janssen booster. J&J /Janssen boosters can be considered for:* i) those who had a severe reaction to Moderna or Pfizer-BioNTech vaccine ii) those who would otherwise remain unvaccinated due to limited access to Pfizer-BioNTech or Moderna vaccines iii) those who want to get the J&J/Janssen booster despite safety recommendations	AD26.COV2-S	✓	
Moderna	mRNA vaccine	2 doses, 28 days apart	1 dose, ≥ 6 months after final dose in primary series (for Moderna and Pfizer-BioNTech recipients) OR ≥ 2 months after vaccination with J&J/Janssen	SpikeVax (mRNA-1273)	✓	
Pfizer-BioNTech	mRNA vaccine	2 doses, 21 days apart	1 dose, ≥ 6 months after final dose in primary series (for Moderna and Pfizer-BioNTech recipients) OR ≥ 2 months after vaccination with J&J/Janssen	Comirnaty (BNT162b2)	✓	
Sinopharm	Inactivated, whole virus	2 doses, 21 days apart	n/a	BIBP	✓	✓
Sinovac	Inactivated, whole virus	2 doses, 14-28 days apart	n/a	CoronaVac	✓	✓

^a^On November 29, 2021, the CDC recommended the following booster dose guidance for non-immunocompromised adults, aged 18 years and older

In this project, we assessed IgG antibodies targeting the SARS-CoV-2 spike and nucleocapsid proteins (anti-S and anti-N, respectively). Table 1 summarizes which antibodies each COVID-19 vaccine is expected to elicit; SARS-CoV-2 infection should elicit both an anti-S and anti-N response. These distinctions are important for understanding our results. For instance, a student vaccinated with an mRNA vaccine, but with a detectable anti-N response, is likely to have been previously infected with SARS-CoV-2.

We collected student blood specimens (5mL–8mL) at a designated phlebotomy site on campus during both the September and December project time points. We stored all collected blood in 10mL serum separator vacutainer tubes for up to 3 hours before transport to the UW-affiliated laboratory. Specimens were processed through a centrifuge at 3500 rpm for 10 minutes and serum supernate was extracted and stored at 4°C, before transport on dry ice to CDC’s laboratory for quantitative serologic testing.

Anti-S and anti-N antibody levels were detected using Meso Scale Discovery V-PLEX SARS-CoV-2 Panel 2 (IgG) kits, a highly sensitive serologic assay for detection of anti-SARS-CoV-2 immunoglobulin-G (IgG) antibodies [17, 18]. We tested specimens in duplicate for each IgG target and reported average values as the WHO-standardized binding antibody units per milliliter (BAU/mL) [19]. Based on manufacturer recommendation, we established thresholds of positivity at 17.66 BAU/mL for anti-S and 11.8 BAU/mL for anti-N targets. These quantitative serologic data were then linked to data reported on the student survey.

We categorized students as previously positive for COVID-19 if (i) on the questionnaire, they self-reported a previous positive COVID-19 test dated before their blood draw; or (ii) they provided sera which resulted with anti-S and anti-N IgG levels above respective thresholds of positivity and had not reported a positive COVID-19 test or had not been vaccinated with an IWV vaccine.

Students were designated as fully vaccinated, boosted, partially vaccinated, or unvaccinated based on CDC guidance for adults without an immunocompromising condition aged ≥18 years. Fully vaccinated students were those with a blood draw date >14 days after receipt of the final COVID-19 vaccine in their recommended primary series. We categorized students as boosted if their blood draw date was ≥7 days after receipt of a COVID-19 booster dose (or any vaccine dose received after completion of a primary series). Partially vaccinated students were those with ≥1 dose of a COVID-19 vaccine who did not meet the fully vaccinated definition. Unvaccinated students were those who had not received any doses of a COVID-19 vaccine at time of blood draw. In categorizing students, we accounted for a student’s immunocompromised status because this increased the number of vaccine doses recommended for primary series completion [20].

### Statistical analysis

Quantitative IgG levels for anti-S and anti-N were transformed to log(10) scale and then summarized by type of primary COVID-19 vaccine received and binary COVID-19 booster status (boosted or unboosted). IgG values were summarized using scatterplots and boxplots. We used notched boxplots to assess statistical significance of differences in median IgG values associated with vaccine profiles, from which we excluded students previously positive for COVID-19 [21, 22].

We used 2 mixed-effects linear regression models to estimate the association between anti-S IgG BAU/mL and either (i) time since receipt of final vaccine in primary series or (ii) time since receipt of booster dose. These models were only applied to students without prior history of positive COVID-19 tests and those without any reported immunosuppressed condition. The model was additionally adjusted for self-reported sex. Because some students participated in both September and December and contributed multiple observations which are not independent, we included a random effect for regression intercept based on the student identification number. We visually assessed Cook’s Distance plots (standardized residuals vs leverage) to determine any points with outsized influence. Identified outliers with excess influence were excluded from regression model calculations but were still presented in scatter plots.

To assess whether primary vaccine platform (e.g., mRNA vs IWV) modified the association between time and anti-S levels, we also ran the same two models above using an interaction term between primary vaccine type and time since vaccination. ANOVA methods were used to assess the change in model fit, with and without the interaction term. R (version 4.1.1) was used to analyze data and produce all figures [23].

### Ethical considerations

Students aged 18 years and older provided written informed consent prior to participation in the project. For students <18 years who were interested in participating, project staff obtained informed written consent and additionally received verbal informed consent from at least one of the student’s parents and/or guardians. Students were not compensated for participating in this project. Students were provided with binary serology results (positive or negative) for both anti-S and anti-N antibodies based on serology testing at the Wisconsin State Laboratory of Hygiene before sera were transported to CDC [24, 25]. Students were also provided with a guide to interpreting their results. This activity was reviewed by CDC and was conducted consistent with applicable federal law and CDC policy.^1^

## Results

In total, 356 students participated in serology testing during the 2021 fall semester (Table 2). Average age was 19.5 years (standard deviation = 2.0 years). Most students were female (56.2%), White (55.6%), non-Hispanic or Latino (86.5%), and freshmen (59.0%). Among December participants, 25.0% (43/172) participants were from countries outside of the United States; the 3 most common countries were China, India, and South Korea.

**Table 2:** Characteristics of students participating in a COVID-19 serology investigation — fall academic semester 2021, Wisconsin

	**Participation Month**						**Overall**	
	***September only***		***September and December***		***December only***			
**Total number of participants, No. (%)**	184	100%	39	100%	133	100%	356	100%
**Age (yrs), mean (sd)**	19.1	1.3	20	1.9	20	2.6	19.5	2
**Sex, No. (%)**								
Female	97	52.7%	25	64.1%	78	58.6%	200	56.2%
Male	77	41.8%	13	33.3%	53	39.8%	143	40.2%
Nonbinary or other	2	1.1%	0	--	1	0.8%	3	0.8%
Refused or missing	8	4.3%	1	2.6%	1	0.8%	10	2.8%
**Race, No. (%)**								
Asian	79	42.9%	11	28.2%	38	28.6%	128	36.0%
Black	6	3.3%	0	--	1	0.8%	7	2.0%
White	82	44.6%	27	69.2%	89	66.9%	198	55.6%
Other	8	4.3%	0	0%	3	2.3%	11	3.1%
Refused or missing	9	4.9%	1	2.6%	2	1.6%	12	3.4%
**Ethnicity, No. (%)**								
Hispanic or Latino	10	5.4%	0	--	5	3.8%	15	4.2%
Non-Hispanic or Latino	149	81.0%	38	97.4%	121	91.0%	308	86.5%
Refused or missing	25	13.6 %	1	2.6%	7	5.3%	33	9.3%
**Class, No. (%)**								
Freshman	134	72.8%	19	48.7%	57	42.9%	210	59.0%
Sophomore	26	14.1%	9	23.1%	43	32.3%	78	21.9%
Junior	6	3.3%	4	10.3%	16	12.0%	26	7.3%
Senior	6	3.3%	6	15.4%	10	7.5%	22	6.2%
Graduate and above	5	2.7%	1	2.6 %	6	4.5%	12	3.4%
Refused or missing	7	3.8 %	0	--	1	0.8%	8	2.2%
**Immunocompromised condition, No. (%)**^**a**^								
Yes	4	2.2%	0	--	6	4.5%	10	2.8%
Refused or missing	7	3.8%	0	--	1	0.8%	8	2.2%
**Home country**								
United States	n/a	--	29	74.4%	99	74.4%	n/a	--
China	n/a	--	6	15.4%	16	12.0%	n/a	--
India	n/a	--	0	--	8	6.0%	n/a	--
South Korea	n/a	--	0	--	4	3.0%	n/a	--
Other^b^	n/a	--	4	10.3%	5	3.8%	n/a	--
Missing	n/a	--	0	--	1	0.8%	n/a	--

^a^Presence of HIV/AIDS, recent organ transplant, or other immunocompromising condition

^b^Includes Bangladesh (Sep/Dec – 0, Dec – 1), Germany (Sep/Dec – 1), Indonesia (Sep/Dec – 1), Kazakhstan (Dec – 1), Malaysia (Dec – 1), Pakistan (Sep/Dec – 1), United Arab Emirates (Sep/Dec – 1), and Vietnam (Dec – 1)

In September, 223 students provided blood samples for serologic testing (Table 3). At time of blood draw, 206 students (92.4%) were fully vaccinated, 3 (1.3%)  had received a booster dose, 5 (2.2%) were partially vaccinated, and 12 (5.4%) were unvaccinated. Most September participants had received mRNA vaccinations (30.9% Pfizer-BioNTech vaccine and 23.8% Moderna vaccine); the third and fourth most common vaccine types reported were IWV vaccines, Sinovac and Sinopharm (18.4% and 13.0%, respectively). The 3 students with self-reported booster doses had all received a full primary series of Sinopharm vaccine doses outside the United States and had received a dose of the Pfizer-BioNTech vaccine when they arrived on the UW campus.

**Table 3:** Vaccination status of students participating in COVID-19 serology investigation — fall academic semester 2021, Wisconsin

	**September**		**December**	
**Total participants**	223	100%	172	100%
**Status, No. (%)**^**a**^				
Unvaccinated	12	5.4%	10	5.8%
Partially vaccinated	5	2.2%	4	2.3%
Fully vaccinated	203	91.0%	109	63.4%
Boosted	3	1.3%	49	28.5%
**Primary series received, No. (%)**				
CoviShield (AstraZeneca)	3	1.3%	5	2.9%
Covaxin	0	--	2	1.2%
Johnson & Johnson/Janssen (J&J/Janssen)	16	7.2%	11	6.4%
Moderna	53	23.8%	50	29.1%
Pfizer-BioNTech	69	30.9%	72	41.9%
Sinopharm (BIBP)	29	13.0%	12	7.0%
Sinovac (Coronavac)	41	18.4%	10	5.8%
Unvaccinated	12	5.4%	10	5.8%
**Detailed primary series plus booster, No. (%)**				
Covaxin plus J&J/Janssen	0	--	1	0.6%
J&J/Janssen plus Moderna	0	--	4	2.3%
J&J/Janssen plus Pfizer-BioNTech	0	--	1	0.6%
Moderna plus Moderna	0	--	12	7.0%
Moderna plus Pfizer-BioNTech	0	--	1	0.6%
Pfizer-BioNTech plus Moderna	0	--	1	0.6%
Pfizer-BioNTech plus Pfizer-BioNTech	0	--	23	13.4%
Sinopharm plus Pfizer-BioNTech	3	1.3%	4	2.3%
Sinovac plus Moderna	0	--	1	0.6%
Sinovac plus Pfizer-BioNTech	0	--	1	0.6%
Not boosted	220	98.7%	123	71.5%
**Previous COVID-19 positive, No. (%)**				
No	185	83%	128	74.4%
Yes, self-report	32	14.3%	37	21.5%
Yes, based on serology but not self-reported^b^	6	2.7%	7	4.1%

^a^Fully vaccinated students were those with a blood draw date more than 14 days after receipt of the final COVID-19 vaccine in their primary series. Boosted students were those with a blood draw date at least 7 days after receipt of a booster dose of a COVID-19 vaccine (or any vaccine dose received after completion of a primary series). Partially vaccinated students were those with at least 1 dose of a COVID-19 vaccine who did not meet the fully vaccinated definition. Unvaccinated students were those who had not received any doses of a COVID-19 vaccine at time of blood draw. Immunocompromised status was considered when categorizing students by vaccination status.

^b^Sera was resulted with anti-S and anti-N IgG antibody levels above the threshold of positivity (>17.66 BAU/mL and >11.8 BAU/mL, respectively) and no history of an inactivated whole virus vaccine.

In December, blood samples were collected from 172 students, including 39 (22.7%) who also participated in September. At that time of blood draw, 49 (28.5%) reported having received a booster dose and 10 (5.8%) reported being unvaccinated. Similar to September participants, Pfizer-BioNTech and Moderna mRNA vaccines were the 2 most reported primary series (41.9% and 29.1%, respectively).

In September and December, 17.0% (38/223) and 25.6% (44/172) of students were previously positive for COVID-19, respectively (Table 3). In September, 6 students (3 Moderna vaccine recipients, 2 Pfizer-BioNTech vaccine recipients, and 1 unvaccinated student) had positive anti-S and anti-N levels but reported no previous positive COVID-19 testing (6/38, 15.8%). This proportion of unrecognized COVID-19 infections was comparable among December samples (7/44, 15.9%); in addition to 1 student who also participated in September (Moderna vaccine recipient), 6 December students had positive anti-S and anti-N levels (2 Moderna vaccine recipients and 4 Pfizer-BioNTech vaccine recipients) but had reported no previous positive COVID-19 testing.

**Fig. 1:**
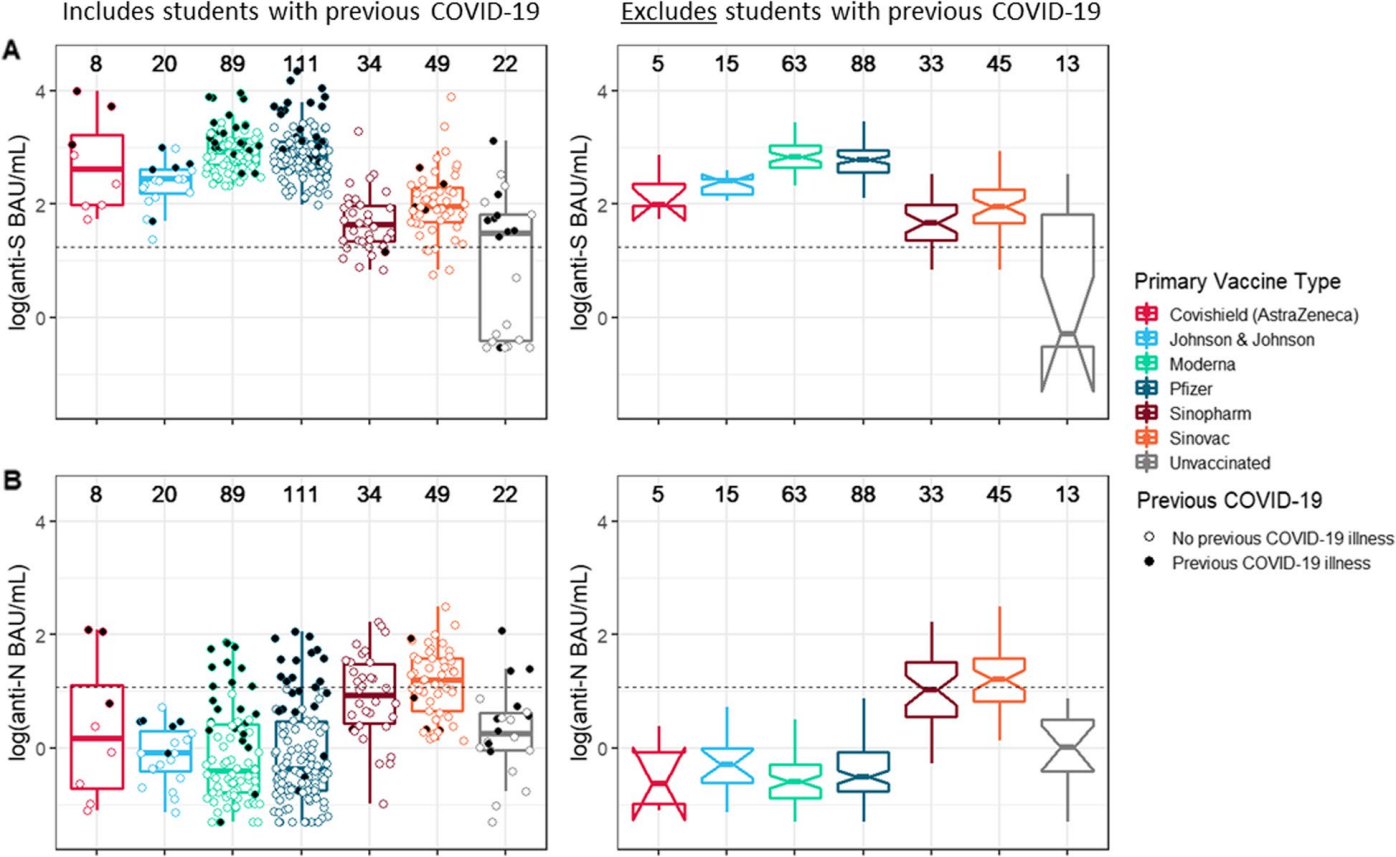
Distribution of anti-SARS-CoV-2 IgG antibody levels from sera samples (N = 333) collected from a sample of university students (*N* = 306) who had only completed a primary series of a COVID-19 vaccine or were unvaccinated — fall academic semester 2021, Wisconsin. Top row, **A:** antibody levels targeting the spike protein of SARS-CoV-2 (anti-S). Bottom row, **B:** antibody levels targeting the nucleocapsid of SARS-CoV-2 (anti-N). Within each row, on the left, a scatterplot—shaded according to previous COVID-19 status—and overlaid boxplot displays antibody levels in WHO-standardized binding antibody units per milliliter (BAU/mL), log(10) scale. On the right, notched boxplots are presented, reflecting the 95% confidence intervals around the median value; these only reflect data from students without previous COVID-19. Total datapoints per category are included above each vaccine group. Horizontal dashed lines indicate the assay threshold of positivity. One student (with receipt of Covaxin primary series) not presented

Among students who had only completed a primary series and without previous positive COVID-19 test, Moderna and Pfizer-BioNTech vaccinations were associated with the highest median values of anti-S IgG (2.83 and 2.78 log [BAU/mL], respectively; Fig. 1A, Table 4). The calculated 95% CIs (boxplot notches) indicated that these median anti-S values for Moderna and Pfizer-BioNTech vaccinees were significantly higher than those associated with J&J/Janssen, Sinopharm, and Sinovac vaccines (2.41, 1.66, and 1.95 log [BAU/mL], respectively). Median anti-N values were significantly higher among IWV vaccine recipients than those associated with mRNA- or vector-based vaccines (Fig. 1B, Table 4).

**Table 4:** Summary of serology results by primary series and booster dose status — fall academic semester 2021, Wisconsin

Vaccine profile			**Anti-S**			**Anti-N**				**Anti-S**		**Anti-N**	
	Primary series received	N	Median		95% CI	Median	95% CI		Vaccine platform	Median	95% CI	Median	95% CI
Unvaccinated		13	-0.29		(-0.52, 2.04)	0.0	(-0.77, 0.53)		n/a	--	--	--	--
**Fully vaccinated with primary series only**													
CoviShield (AstraZeneca)		5		1.99	--	-0.63	--	**Vector-based**		2.35	(2.06, 2.44)	-0.32	(-0.70, -0.07)
Johnson & Johnson		15		2.41	(2.13, 2.45)	-0.29	(-0.70, 0.09)						
Moderna		63		2.83	(2.71, 2.90)	-0.59	(-0.77, -0.50)	**mRNA**		2.80	(2.74, 2.86)	-0.56	(-0.61, -0.46)
Pfizer		88		2.78	(2.66, 2.87)	-0.51	(-0.62, -0.36)						
Covaxin		1		2.01	--	0.50	--	**Inactivated whole virus**		1.87	(1.69, 2.00)	1.10	(0.99, 1.30)
Sinopharm		33		1.66	(1.39, 1.94)	1.02	(0.62, 1.25)						
Sinovac		45		1.95	(1.82, 2.20)	1.21	(1.02, 1.42)						
**Primary series and mRNA booster**													
CoviShield (AstraZeneca)		0		--	--	--	--	**Vector-based**		4.01	--	0.29	--
Johnson & Johnson		5		4.01	--	-0.22	--						
Moderna		8		4.07	(-0.52, 4.31)	0.42	(-1.30, 0.77)	**mRNA**		3.82	(3.61, 3.96)	-0.14	(-0.70, 0.08)
Pfizer		20		3.69	(3.57, 3.88)	-0.47	(-0.98, -0.03)						
Covaxin		1		3.20	--	1.22	--	**Inactivated whole virus**		3.27	--	0.29	--
Sinopharm		7		3.31	--	0.07	--						
Sinovac		2		3.45	--	0.45	--						

*Anti-S *Antibody levels targeting the spike protein of SARS-CoV-2. *Anti-N *Antibody levels targeting the nucleocapsid of SARS-CoV-2. *95% CI *95% confidence interval around the median value for each group, which are not calculated for groups with N≤10. Antibody levels are presented in WHO-standardized binding antibody units per milliliter (BAU/mL), log(10) scale

**Fig. 2:**
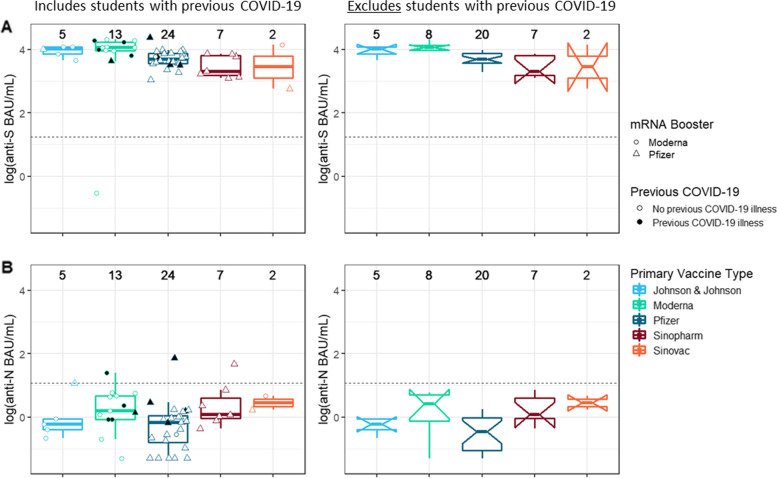
Distribution of anti-SARS-CoV-2 IgG antibody levels from sera samples (*N* = 51) collected from a sample of university students (*N* = 49) with receipt of an mRNA COVID-19 vaccine booster dose — fall academic semester 2021, Wisconsin. Top row, **A:** antibody levels targeting the spike protein of SARS-CoV-2 (anti-S). Bottom row, **B:** antibody levels targeting the nucleocapsid of SARS-CoV-2 (anti-N). Within each row, on the left, a scatterplot—shaded according to previous COVID-19 status—and overlaid boxplot displays antibody levels in WHO-standardized binding antibody units per milliliter (BAU/mL), log(10) scale. On the right, notched boxplots are presented, reflecting the 95% confidence intervals around the median value; these only reflect data from students without previous COVID-19. Total datapoints per category are included above each vaccine group. Horizontal dashed lines indicate the assay threshold of positivity. One student (with receipt of Covaxin primary, J&J/Janssen booster) not presented

Among students boosted with an mRNA vaccine, distribution of anti-S IgG values was more condensed across vaccine types than among students with only primary series receipt (Fig. 2A). Regardless of primary vaccine type, receipt of an mRNA booster dose increased the median anti-S IgG value. For instance, among students without a previous positive COVID-19 test, those who had received Pfizer-BioNTech as a primary series were associated with a median anti-S IgG value not statistically significantly different than those who had received a Sinopharm vaccine primary series (3.69 vs 3.31 log [BAU/mL], respectively). No obvious visual differences existed in IgG level improvement associated with either Moderna or Pfizer-BioNTech mRNA vaccine boosters. No obvious visual differences in median anti-N levels were observed across vaccine types following receipt of an mRNA booster (Fig. 2B).

**Fig. 3:**
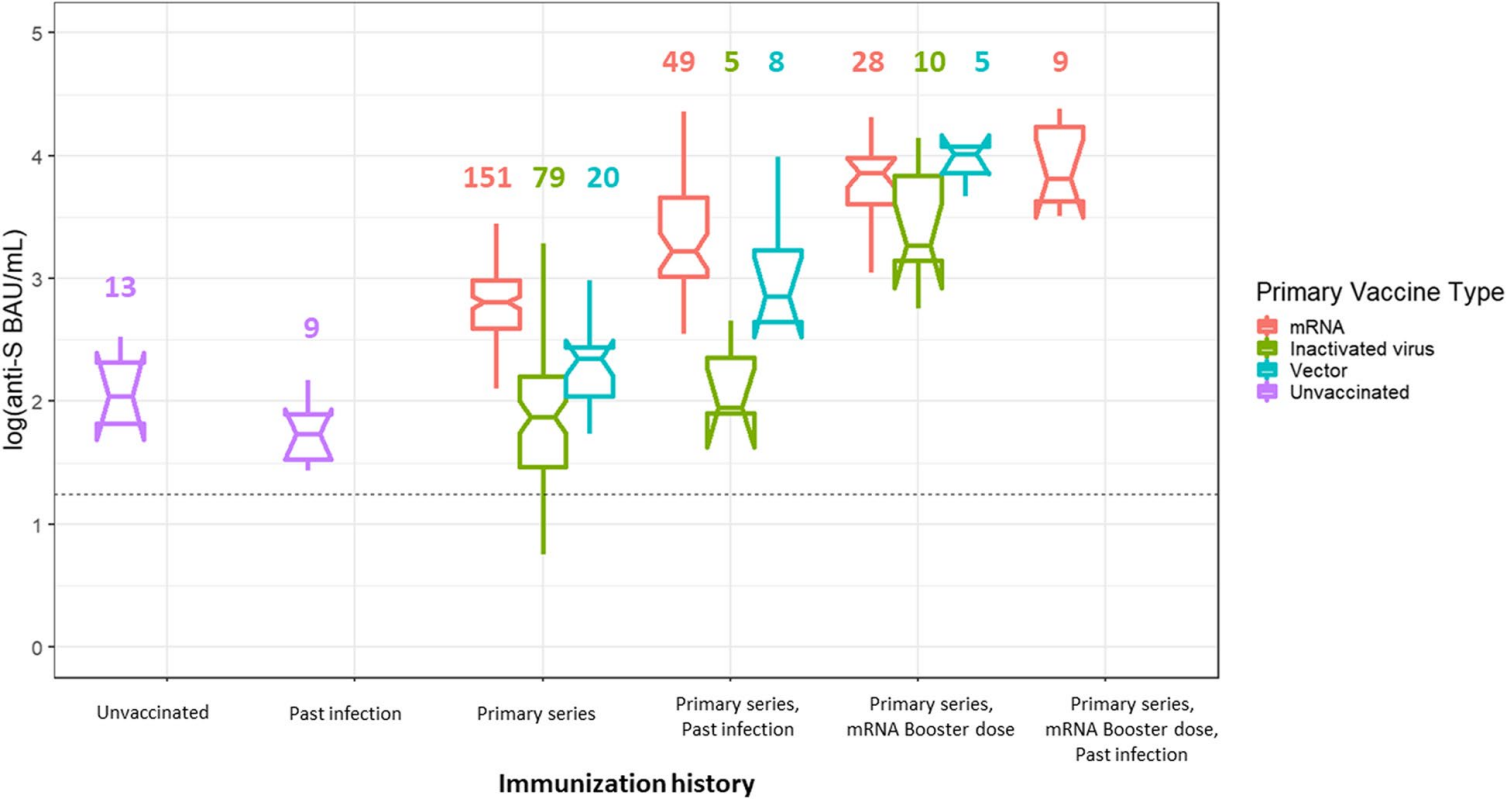
Stratified boxplots representing the distribution of anti-spike antibody levels detected in 393 sera specimen classified by immunization history of student at time of blood draw — fall academic semester 2021, Wisconsin. Boxplots reflect antibody levels targeting the spike protein of SARS-CoV-2 (anti-S) in WHO-standardized binding antibody units per milliliter (BAU/mL), log (10) scale. Notched boxplots are presented, where the width of the notch indicates the 95% CIs around the median value. Horizontal dashed lines indicate the assay threshold of positivity

The reduced difference in anti-S level across vaccines was also apparent when primary vaccine type was collapsed to type of vaccine platform (e.g., mRNA vs IWV vs vector-based; Fig. 3). The median anti-S value for boosted mRNA recipients was not significantly different from that of boosted IWV recipients (3.82 vs 3.27 log [BAU/mL], respectively).

**Fig. 4:**
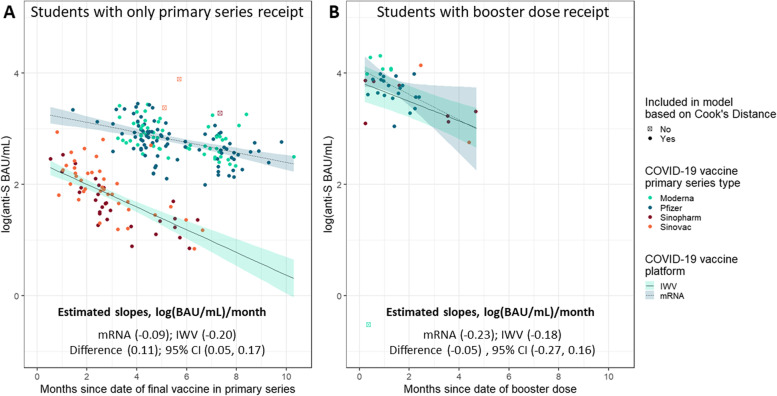
Anti-S IgG antibody levels and multivariate mixed-effects regression model estimates among university students with no history of COVID-19 or immunocompromised status, faceted by booster dose receipt — fall academic semester 2021, Wisconsin. Panels display levels of antibody targeting the spike protein of SARS-CoV-2 (anti-S), in WHO-standardized binding antibody units per milliliter (BAU/mL), log (10) scale. Sex-adjusted, mixed-effects linear regression estimates are superimposed on top of scatter plots with shading reflecting the 95% confidence intervals to estimate anti-S decline associated with primary series vaccine type (e.g., mRNA vs inactivated whole virus, or IWV). Panel **A** displays anti-S values of students with receipt of COVID-19 vaccine primary series across months since completion of primary series. Panel **B** displays anti-S values of students who received an mRNA booster dose across months since receipt of booster dose. Data outliers that were not included in the model based on Cook’s Distance are denoted as a boxed point and were not used to calculate adjusted regression lines

The sex-adjusted, mixed-effects linear regression model indicated a negative association between time since primary series vaccination and anti-S IgG (Fig. 4A). An interaction term between vaccine platform and time since vaccination statistically improved model fit (χ^2^ = 14.7; *P* <.001), indicating a statistically significant difference between mRNA vaccines and IWV vaccines (β for interaction term = 0.11; 95% CI = .05–.17). Anti-S decline over time was also apparent among boosted students (Fig. 4B), however the difference in rate was not significantly different between students with either mRNA or IWV primary receipt. Moreover, our regression model suggested a similar intercept value for anti-S IgG levels associated with both mRNA and IWV vaccines, approximately 3.8 log (BAU/mL). This can be interpreted roughly as the anti-S IgG level at 0 days following mRNA booster injection.

**Fig. 5:**
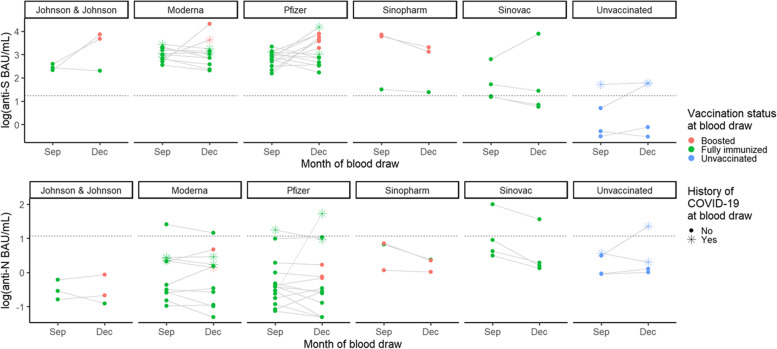
Paired sera results for students who participated during the investigation’s September and December time points — fall academic semester 2021, Wisconsin. Top panel displays the levels of antibody targeting the spike protein of SARS-CoV-2 (anti-S) and the bottom panel displays the levels of antibody targeting the nucleocapsid protein (anti-N), both in WHO-standardized binding antibody units per milliliter (BAU/mL), log (10) scale. Month of blood draw is presented on the x-axis. Sera values are categorized based on previous history of COVID-19 illness (shape) and vaccination status (color) at blood draw. Horizontal dashed lines indicate the assay threshold of positivity

Data from the 39 students who participated in both September and December highlighted a booster-associated increase in anti-S IgG (Fig. 5). In total, 10 students were boosted between the September and December blood draws, all of whom experienced an increase in anti-S IgG levels—5 with an over 10-fold increase (50.0%). Of the 19 students who were not boosted and did not report a positive COVID-19 test between September and December, 17 (89.5%) were associated with declines in anti-S IgG level. An additional 4 students were not boosted, but did report a positive COVID-19 test between blood draws; 2 (50.0%) were associated with increases in anti-S levels.

## Discussion

Among a group of university students, we detected significant differences in median anti-S IgG levels and rates of decline after primary series completion with various COVID-19 vaccines. Median anti-S levels were approximately 10-times higher for both Pfizer-BioNTech and Moderna mRNA vaccine recipients, compared with either Sinopharm or Sinovac (IWV) vaccine recipients; additionally, the negative association between anti-S levels and time since primary series completion was significantly more pronounced among IWV recipients, compared with students who received an mRNA vaccine primary series. These serologic findings support previous work that compared serologic outcomes across vaccine types, which demonstrated mRNA vaccines are consistently associated with a more robust anti-S IgG response [26–29].

More importantly, among IWV primary series recipients, an mRNA booster dose increased anti-S antibody response to levels comparable with mRNA primary series recipients. In other words, an mRNA booster dose appeared to increase anti-S IgG to comparable levels regardless of the type of COVID-19 vaccine primary series. COVID-19 mRNA booster doses could be considered an important supplement for people administered non-mRNA formulations.

A substantial proportion of UW students reported receipt of a COVID-19 primary series at the start of the 2021 fall semester. That said, students who completed a primary series outside the United States might have been less protected than students who completed a primary series in the United States because of decreased vaccine-associated antibody response—with regards to both an initial response and waning over time. Certainly, the direct and indirect protection afforded by any COVID-19 vaccine is better than no vaccination. However, our work found a significantly lower antibody response and a more pronounced negative association between anti-S levels and time since IWV primary series completion, compared with an mRNA primary series. Thus, despite full vaccination status, international students with IWV-receipt might have been at higher risk for COVID-19 infection and transmission during the fall 2021 semester. At UW, where international students make up an estimated 13.5% of the student body [9], COVID-19 risk was potentially magnified considering that international students are more likely to socialize with co-national peers [30]. Through the lens of vaccine equity, public health experts and policy makers working in populations of differentially vaccinated persons should take care to recognize subgroups who might benefit from additional vaccination.

We found that an mRNA booster dose helped to normalize antibody levels throughout the student population. Not only did we find similar anti-S levels among IWV and mRNA vaccine recipients after an mRNA booster dose, but our mixed-effects regression model demonstrated that post-booster, anti-S rates of decline were statistically similar between both IWV and mRNA primary series type. Although a threshold of protection associated with a certain anti-S IgG level remains unclear [31], evidence indicates that anti-S IgG levels of Sinopharm or Sinovac primary series recipients would drop below that threshold before Moderna or Pfizer-BioNTech primary series vaccine recipients. A difference in this time-to-threshold would likely be less pronounced among an mRNA-boosted group of students.

Though not a randomized clinical trial, our real-world findings add to a growing body of evidence which supports the use of heterologous boosting (or boosting with a COVID-19 vaccine type different from that of the primary series vaccine type). In the United States, researchers considering only J&J/Janssen, Moderna, and Pfizer-BioNTech vaccine combinations found immunogenicity resulting from heterologous boosting comparable, or even more robust, than that of homologous boosting [32–34]. An inactivated-to-mRNA prime-boosting regimen (similar to many students in our project) has specifically been shown to be safe and, as Zuo *et al.*note, a regimen that “strongly augments” the immune response [35, 36]. Among Sinovac recipients in a large observational study in Chile, though both homologous and heterologous booster doses resulted in strong protection against COVID-19 outcomes (including against symptomatic COVID-19, critical care, and death), those who were boosted with Pfizer-BioNTech were associated with a significantly greater vaccine effectiveness, compared with those who were boosted with Sinovac (adjusted vaccine effectiveness against symptomatic COVID-19 = 96.5% and 78.8%, respectively; *P*<.05) [37]. We were unable to assess homologous boosting with IWV; given U.S. public health guidance[38], no students had the option to be boosted with Sinovac nor Sinopharm.

Given these and other data, CDC guidelines allow for a "mix-and-match" booster dose strategy for Pfizer and Moderna COVID-19 vaccines; the use of the J&J/Janssen COVID-19 vaccine is recommended in certain limited situations [38]. CDC booster guidance also addresses vaccination of persons who received ≥1 COVID-19 vaccines outside of the United States, albeit in an online appendix [39]. Information contained within this type of guidance is important to promote, especially in populations like that of our investigation with a diverse range of nationalities. This likely applies to numerous U.S. university campuses. More broadly, this type of policy would also be applicable to any population with residents that were vaccinated against COVID-19 outside the United States (e.g., work visa holders, migrant workers, refugees, or asylees).

The findings in this analysis are subject to at least 4 methodological limitations. For one, the level of IgG antibodies associated with protection against COVID-19 outcomes remains unclear [27, 40–43]. In part, this is because protection against COVID-19 is associated with other immunologic responses outside of just anti-S and anti-N IgG, such as cell-mediated immunity. This is also due to the substantial number of commercially available serologic assays which limit the comparability of serostudies [44, 45]. Our results followed WHO guidance and reported our findings in the standardized unit (BAU/mL) for anti-SARS-CoV-2 antibody levels; however, as Perkmann *et al*found, use of BAU/mL might not overcome the systematic differences across the many available anti-SARS-CoV-2 assays [46]. Second, interpretation of our assay results is also challenged by the fact that this was an investigation using real-world data among a convenience sample of students, which limits generalizability. This also meant that small numbers hampered our ability to confidently assess antibody levels among specific vaccine series groupings (e.g., only one student had received a Sinovac primary plus mRNA booster). Third, we relied on self-reported information, which might undercount the true number of previous COVID-19 cases in our participant population. Previous infection would influence the anti-S and anti-N IgG levels observed among participants; whereas we were able to identify a limited number of students with unrecognized previous infection, for serologic reasons (Table 1) we could only correct for unknown previous infection among mRNA or vector-based vaccine recipients. Fourth, these results reflect a period when Delta was the dominant circulating variant strain in Wisconsin. How future mutations in the SARS-CoV-2 virus will affect IgG binding to mutated antigens is unclear [47]. Thus, we cannot say whether the infection-associated rise in antibodies that we observed will remain the same as the SARS-CoV-2 virus continues to mutate and new variants emerge. Variant emergence also complicates any discussion concerning correlates of protection and to what degree a certain antibody level protects an individual from a given COVID-19 outcome. Thus, the associated rise in anti-S IgG following an mRNA booster dose, and the benefit of doing so among IWV vaccinated persons, could offer different protection as new variants arise.

Overall, our study offers more data suggesting that IWV vaccine types result in a lower immune response and faster decline over time, compared with mRNA vaccines. International students compose a U.S. population subgroup that might be at higher risk for SARS-CoV-2 infection and transmission given lower antibody levels because of type of vaccines received. One way to better protect such a subgroup, and indeed all U.S. residents with only receipt of an IWV primary series, is evidenced in our findings. Namely, heterologous vaccine boosting with mRNA doses might significantly improve the anti-SARS-CoV-2 antibody levels among persons who received COVID-19 IWV vaccines outside of the United States.

## Data Availability

The datasets used and analyzed during the current study are available from the corresponding author on reasonable request.

## Abbreviations

WHO: World Health Organization
IWV: Inactivated whole virus
UW: University of Wisconsin – Madison
BAU/mL: Binding antibody units per milliliter
CDC: US Centers for Disease Control and Prevention
Anti-S: Anti-spike
Anti-N: Anti-nucleocapsid

## Footnotes

1 Activity was determined to meet the requirements of public health surveillance as defined in 45 CFR 46.102(l)(2)This activity was reviewed by CDC and was conducted consistent with applicable federal law and CDC policy.§§ See e.g., 45 C.F.R. part 46.102(l)(2), 21 C.F.R. part 56; 42 U.S.C. §241(d); 5 U.S.C. §552a; 44 U.S.C. §3501 et seq.
